# Open questions: CRISPR biology

**DOI:** 10.1186/s12915-018-0565-9

**Published:** 2018-09-24

**Authors:** Eugene V. Koonin

**Affiliations:** National Center for Biotechnology Information, National Library of Medicine, Bethesda, MD 20894 USA

## Abstract

CRISPR-Cas systems, the purveyors of adaptive immunity in archaea and bacteria and sources of the new generation of genome engineering tools, have been studied in exquisite molecular detail. However, when it comes to biological functions, ecology, and evolution of CRISPR-Cas, many more intriguing questions remain than there are answers.

CRISPR-Cas, the adaptive immunity system of bacteria and archaea, is the source of the molecular tools that, during the last 5 years, have revolutionized genome engineering. Thanks to this extraordinary practical importance, the mechanisms of CRISPR-Cas activities have been studied in almost unprecedented structural and biochemical detail [1]. Although much remains to be determined—in particular, with regard to the mechanisms of adaptation, the first key stage of the CRISPR response, during which pieces of foreign DNA are incorporated into a CRISPR array—I believe it is a fair claim that we are approaching a thorough understanding of CRISPR-Cas at the molecular level.

To me, however, the real beauty of CRISPR-Cas is that, apart from being so useful, these systems present a plethora of intriguing, fundamental biological problems that we are only now starting to grasp, let alone solve. I briefly discuss some of these open problems below.

1. Why are CRISPR-Cas systems so unevenly and sparsely—compared to other defense systems—distributed in the microbial world? Virtually all archaea, most especially hyperthermophiles, have CRISPR-Cas, but only about one-third of bacteria do. This is in sharp contrast to some other defense systems, such as restriction-modification, which is virtually ubiquitous among prokaryotes except for certain parasitic bacteria. What is special about archaea and/or hyperthermophiles that they are so “fond” of CRISPR-Cas? Do these organisms live in the range of virus diversity and abundance in which adaptive immunity is particularly advantageous, as suggested by some mathematical models of CRISPR-virus coevolution? Or are there molecular mechanisms that make CRISPR-Cas indispensable for them? Solving this problem will shed new light on CRISPR biology.
2. A series of questions orthogonal to the first one: why are class 1 CRISPR-Cas systems (those with multisubunit protein complexes, known as effectors, involved in CRISPR RNA processing and target cleavage) so much more prevalent among prokaryotes than class 2 systems, those with single-protein effectors [2]? Why are class 2 systems virtually absent in archaea and completely non-existent in hyperthermophiles? And, among the class 2 systems, why is type II orders of magnitude more common than types V and VI? Our current inability to answer these questions indicates that we remain ignorant of fundamental aspects of CRISPR-Cas functions.
3. What is the cost of CRISPR-Cas systems for the microbes that carry them? How common and how dangerous is autoimmunity? These questions are certainly linked to the first one on my list, on the uneven spread of CRISPR-Cas immunity among prokaryotes. The current message is mixed, with little—if any—cost identified in direct experiments with CRISPR-carrying bacteria in the absence of infection, but substantial autoimmunity detected for at least some CRISPR-Cas systems.
4. A related group of questions: how important and how efficient is self vs non-self discrimination by CRISPR-Cas systems? Are these systems wasteful such that the majority of cells in a CRISPR-carrying microbial population die of autoimmunity, but the resistance of the surviving few to virus infection outweighs the deleterious effect of autoimmunity? Again, the current results are mixed. Mechanisms for self vs non-self discrimination seem to exist in at least some CRISPR-Cas systems, but do not appear to be particularly strict, that is, involve preference for actively replicating or transcribed DNA [3]. The answers to these questions also pertain to the more general assessment of CRISPR-Cas immunity as an evolutionary phenomenon: is this a genuine Lamarckian-type mechanism of direct adaptation in response to an environmental cue or does selection still play a key role?
5. What is the balance between CRISPR-Cas activity and horizontal gene transfer (HGT)? HGT is the key process of evolution in prokaryotes, the main route of functional innovation and adaptation, and the mechanism for purging deleterious mutations. Defense mechanisms potentially can interfere with HGT, and the highly efficient CRISPR-Cas systems could be particularly detrimental in this regard. The available data are somewhat contradictory. It has been shown that CRISPR-Cas systems indeed prevent acquisition of antibiotic resistance plasmids by certain bacteria [4]. Counter-intuitively, however, they appear to stimulate rather than abrogate gene transduction by bacteriophages [5]. It seems possible that the effects of CRISPR-Cas on different HGT mechanisms genuinely differ. Revealing the relationships between CRISPR-mediated immunity and HGT is important for understanding the actual role of CRISPR-Cas in microbial biology.
6. What is the relationship between CRISPR-Cas, on the one hand, and programmed cell death and microbial dormancy, on the other hand? Do CRISPR-Cas systems switch to dormancy or programmed cell death induction when immunity fails? The appearance of a paradox notwithstanding, it is becoming increasingly clear that programmed cell death is common among unicellular life forms, both eukaryotes and prokaryotes. Can autoimmunity be considered a form of altruistic suicide? Many CRISPR-Cas systems encode homologs of prokaryotic toxins, and some evidence of CRISPR-induced programmed cell death has been reported. It has been proposed that CRISPR-Cas systems “make decisions”, on the basis of the level of genotoxic stress, to execute either the immunity or the altruistic suicide (or dormancy induction) program [6]. This hypothesis awaits experimental testing.
7. How common are non-defense, regulatory, and signal-transduction functions of CRISPR-Cas systems?
  In many respects, CRISPR-Cas systems are analogous to eukaryotic RNA interference—it was actually this analogy that led to the initial prediction of the CRISPR-Cas function and mechanism. Accordingly, it also has been predicted that CRISPR-Cas systems would have both defense and regulatory roles. So far only a few cases of endogenous gene regulation have been characterized in any detail, the best understood one being, probably, the regulation of quorum behavior and sporulation in the mycobacterium *Myxococcus xanthus* [7]. Given that self-targeting CRISPR spacers trigger deleterious autoimmunity, special mechanisms are required for CRISPR-mediated gene regulation such as partial complementarity or involvement of Cas protein complexes devoid of target cleavage activity. The actual range of regulatory activities of CRISPR-Cas systems remains to be discovered.
8. Are CRISPR-Cas systems important for bacterial virulence?
  Related to the preceding group of questions, comparative genomic analysis demonstrates high prevalence of CRISPR-Cas systems, particularly type II, in bacterial pathogens, and apparent CRISPR-dependent regulation of genes involved in pathogenicity has been demonstrated for several pathogenic bacteria [8]. However, a general assessment of the contribution of CRISPR-Cas to bacterial pathogenicity awaits a thorough study.
9. What are the functions of CRISPR-Cas systems encoded by transposons and plasmids?
  Apart from archaeal and bacterial genomes, CRISPR-Cas systems are encoded by a large family of Tn7-like transposons [9] and by various plasmids [2]. All transposon-encoded and some of the plasmid-encoded CRISPR-Cas are “minimal” variants that lack the enzymes involved in target cleavage and hence are predicted to be involved in non-defense roles. The nature of these functions remains to be elucidated, and might involve RNA-guided transposition [9].
10. What is the ultimate origin (s) of CRISPR-Cas?
  Highly complex functional systems such as CRISPR-Cas must have evolved via multiple intermediate stages. Comparative genomic analyses have yielded some notable clues, revealing, in particular, multiple contributions of various mobile genetic elements, including casposons that are thought to be the ancestors of the CRISPR adaptation modules [10]. However, the origin of the effector module of class 1 CRISPR-Cas systems remains uncertain, and so does the actual pathway of its integration with a casposon. There is hope that further, extensive sequencing of diverse microbial genomes will help identify intermediates of CRISPR-Cas evolution.

I have arbitrarily compiled the open problems in CRISPR biology into “top 10” groups of questions. Clearly, different lists could have equal merits, and perhaps some of the questions above are misconstrued or too narrow. Nevertheless, I hope I have conveyed my message: there is actually much more to learn about CRISPR biology than we already know, and it will be a fascinating journey.
